# Incidence of aphid-transmitted viruses in raspberry and raspberry aphids in Norway and experiments on aphid transmission of black raspberry necrosis virus

**DOI:** 10.3389/fpls.2024.1441145

**Published:** 2024-11-01

**Authors:** Bijaya Sapkota, Nina Trandem, Jana Fránová, Igor Koloniuk, Dag-Ragnar Blystad, Zhibo Hamborg

**Affiliations:** ^1^ Division of Biotechnology and Plant Health, Norwegian Institute of Bioeconomy Research, Ås, Norway; ^2^ Biology Centre CAS, Institute of Plant Molecular Biology, České Budějovice, Czechia

**Keywords:** *Rubus*, black raspberry necrosis virus, raspberry leaf mottle virus, Rubus yellow net virus, raspberry vein chlorosis virus

## Abstract

Raspberry (*Rubus idaeus* L.) is susceptible to aphid-borne viruses. We studied the incidence of four of them – black raspberry necrosis virus (BRNV), raspberry leaf mottle virus (RLMV), raspberry vein chlorosis virus (RVCV), and Rubus yellow net virus (RYNV) – in raspberry plants and aphids in and around Norwegian raspberry crops for three years (2019, 2021, and 2022). Most of the samples were from symptomatic plants. Applying RT-PCR, 274 leaf samples and 107 aphid samples were analyzed. All four viruses were found, but BRNV dominated: it was detected in 93% of the 178 leaf samples with virus and was the only virus that occurred more frequently as a single infection than in co-infections with the other viruses. The old cv. Veten had the highest virus incidence (97%) among the sampled plants, followed by uncultivated raspberry in the boundary vegetation (82%). All aphids identified were *Amphorophora idaei* and *Aphis idaei.* BRNV and/or RLMV was detected in 27% of the aphid samples. Notably, BRNV was detected in 30% of *A. idaei* samples, a species not known as a BRNV vector. In subsequent transmission experiments we found that although *A. idaei* can acquire BRNV within one hour, it did not transmit the virus to healthy raspberry plants. In contrast, *Am. idaei*, a known BRNV vector, was able to acquire the virus within one minute and transmit it within one hour of inoculation. Our study will improve the identification and management of BRNV.

## 1 Introduction

Red raspberry (*Rubus idaeus* L.), an economically important perennial crop, reached global production of 886538 tons in 2021 (FAO, 2022). Its popularity has increased significantly, especially in Europe and North America, due to its high nutritional, dietary and medicinal values. Norway is one of the raspberry producing countries in Europe, with an annual production of 1798 tons in 2021 (FAO, 2022). In Norway, the prime area for growing raspberries is in the western part, specifically within the fjord district of Sogn og Fjordane, now part of Vestland county (Bøthun and Heiberg, 2004). This region benefits from an ideal climate for raspberry production, contributing to its prominence in cultivation. Raspberry cv. Veten was the cornerstone cultivar for more than 30 years, mainly serving the processing industry with its robust characteristics (Haffner et al., 2002; Fotirić Akšić et al., 2022). However, the introduction of cv. Glen Ample in 1996 marked a significant shift, as this Scottish-bred variety supplanted cv. Veten due to its high yield with large fruit and excellent quality at high latitudes, flourishing even in the northern reaches of Brønnøysund in Nordland (65° N) (Heiberg et al., 2002; Bøthun and Heiberg, 2004). Recently, the cv. Glen Mor, bred by the James Hutton Institute in 2020, has raised the interest of Norwegian farmers, mainly due to its *Phytophthora* resistance (https://www.huttonltd.com/services/plant-varieties-breeding-licensing/raspberry/glen-mor).

Raspberry plants are vulnerable to a host of pathogens, particularly viruses (Martin et al., 2013). Till now, 24 plant viruses from different families and genera are known to infect raspberry (Tan et al., 2022; Koloniuk et al., 2023; Lenz et al., 2024). Among them, aphid-transmitted viruses, such as black raspberry necrosis virus (BRNV, *Sadwavirus rubi*), raspberry leaf mottle virus (RLMV, *Closterovirus macularubi*), and Rubus yellow net virus (RYNV, *Badnavirus reterubi*), are important and cause raspberry mosaic disease (RMD) when they occur as mixed infections (Converse, 1987; Alford, 2007). Yield losses due to the combined effect of these viruses in some red raspberry cultivars can be significant, ranging from 11 to 39% in different regions (Converse, 1963; Freeman and Stace-Smith, 1970). Individually, these viruses may not exhibit distinct symptoms in red raspberry (*R. idaeus* L.) cultivars (Jones and Jennings, 1980; Martin et al., 2013). For instance, BRNV may cause apical necrosis in shoots of the indicator species *R. henryi* and *R. occidentalis* (Jones and Jennings, 1980), while RLMV induces chlorotic leaf spots and mosaics in *R. idaeus* cv. Malling Landmark, and RYNV causes net-like chlorosis along veins in *R. occidentalis* cv. Munger (Stace-Smith, 1955a; Martin et al., 2013). Another aphid-borne virus, raspberry vein chlorosis virus (RVCV, genus *Rhabdovirus*), induces a yellow net pattern in most cultivars (Martin et al., 2013). The spread of viruses is often unintentionally facilitated by farmers, such as using infected planting material or overlooking slight symptoms, highlighting the importance of efficient detection and production of healthy planting material (Converse, 1987; Tatineni and Hein, 2023).

All viruses transmit efficiently through vegetative propagation techniques, with additional routes of transmission including carriers such as seeds, pollen, and insects such as aphids (Dietzgen et al., 2016). In Europe, the large European raspberry aphid, *Amphorophora idaei* (or *Am. rubi idaei*) is the primary vector transmitting BRNV, RLMV, and RYNV, while the small European raspberry aphid, *Aphis idaei* transmits RVCV (Martin et al., 2013; Tan et al., 2022).

Viral infections, facilitated by vector dispersal strategies, pose a significant challenge once established. The use of virus-free plant material is thus crucial for disease control (Wang et al., 2022a, Wang et al., 2022b; Bettoni et al., 2024). Studying virus occurrence and vector distribution on commercial farms is necessary to establish disease management strategies. The main objective of this study was to assess the relative distribution of aphid-transmitted viruses in symptomatic raspberry and aphids on such plants in commercial raspberry farms across the most important production area in Norway. The secondary objective was to evaluate the capability of *Am. idaei* and *A. idaei* as vectors for BRNV through aphid transmission experiments.

## 2 Materials and methods

### 2.1 Field survey and sample collection

Field sampling was carried out in June and July 2019, 2021, and 2022 in different counties in Norway (in 2020 the covid pandemic led to a break in the sampling). The sample sizes for different years and locations are listed in **Table 1**. The selected plantations were subjected to visual inspection and shoots of raspberry canes with leaves were collected. In the majority of cases, plants displaying virus symptoms were chosen. Additionally, asymptomatic samples with the presence of aphids were also included. A total of 274 samples of raspberry leaves were acquired, consisting predominantly of three cultivars – ‘Glen Ample’, ‘Veten’, and ‘Glen Mor’ - from open fields or polytunnels, and of non-cultivated raspberry plants of unknown variety found within the boundary vegetation of the plantations, referred to as “Wild” cultivars. Furthermore, some cultivated raspberry samples were labeled as “Others”, signifying their unidentified cultivar name. Photographs were taken of all the samples collected to assist in the evaluation of leaf symptoms.

**Table 1:** Locations and number of raspberry leaf samples collected.

Sampling location	Sample size			
	2019	2021	2022	Total
Innlandet	8	0	0	8
Vestfold og Telemark	9	0	0	9
Viken	7	16	27	50
Vestland	39	68	83	190
Agder	0	11	6	17
Total	63	95	116	**274**

The sampling locations are the counties covering the most important raspberry growing areas in Norway, while the sample size indicates the number of raspberry samples collected in three different years (2019, 2021, and 2022).

In 2021 and 2022, any aphids colonizing the sampled leaves were also examined and a total of 107 aphid samples were collected over the two years. Morphological identification was conducted by examining random aphids from each collected sample under a stereo microscope (Lecia MZ72). Only morphologically identified *Am. idaei* and *A. idaei* were collected for molecular identification. The aphids were individually collected and placed in separate 2 ml Eppendorf tubes containing DNA/RNA Shield (Zymo Research, Irvine, CA, USA). For nymphs, 3-5 individuals were collected in a single tube, while for adults, the number was limited to 1-2 per tube.

### 2.2 RNA extraction

Raspberry leaf samples were ground into a fine powder using liquid nitrogen employing a mortar and pestle. Total RNA was then extracted from fresh young leaf tissues (50 mg) using the Norgen Plant/Fungi RNA kit (Norgen Biotek, Thorold, ON, Canada). Extraction was performed according to the manufacturer’s instructions with some modifications, and then elution was performed in 50 μL of RNase-free water. DNase treatment was implemented on the column during the extraction process. In addition, plant samples infected with BRNV, RLMV, RYNV and RVCV and cultivated in the NIBIO greenhouse as positive controls were also subjected to a similar RNA extraction procedure. The amount of RNA was determined using a NanoDrop 1000b spectrophotometer (NanoDrop Technologies, Wilmington, DE, USA) and the extracted RNA was stored at a temperature of −80°C for future use.

Aphid samples collected in the DNA/RNA solution were processed by directly crushing them with a small glass rod and adding 600 μL of TRIzol reagent (Thermo Fisher Scientific, Waltham, MA, USA). The total RNA was extracted using a Direct-zol RNA Miniprep Kit (Zymo Research, CA, *USA*) according to the manufacturer’s instructions. The purified RNA was then stored at −80°C.

### 2.3 RT-PCR and Sanger sequencing

The extracted total RNA was reverse transcribed (RT) using the Superscript IV Reverse Transcriptase kit (Invitrogen, Carlsbad, CA, USA) according to the manufacturer’s guidelines. As an internal amplification control of the plant sample for amplification, RT-PCR was used to amplify mitochondrial NADH dehydrogenase *nad5* mRNA (Menzel et al., 2002). While internal controls of cytochrome c oxidase subunit I-COI (Folmer et al., 1994) were used for aphid samples. For the subsequent virus detection, only samples that passed the internal controls were included and carried out in a 25 µL reaction with Taq DNA polymerase (5U/µL) (InvitrogenTM, ThermoFisher Scientific, USA) according to the manufacturer’s recommendations with 2 µL of cDNA. All primer sequences used in this study as well as their respective amplification conditions are listed in **Table 2**. As a part of quality control, reaction mixtures containing 2 µL of sterile water in place of the cDNA template were used as blank controls, and cDNA templates previously confirmed to be infected with the respective viruses were used as the virus-positive controls. The PCR programs applied to detect all the viruses were as follows: initial pre-denaturation step at 95°C for 2 minutes, followed by 35 cycles of denaturation at 95°C for 30 seconds, annealing at 47 to 60°C for 30 seconds, and extension at 72°C for 45 seconds. A final extension was performed at 72°C for 7 minutes. Each resulting PCR product (10 µL) was subjected to electrophoresis in a 1.2% agarose gel previously stained with SYBR safe DNA stain (Invitrogen, ThermoFisher Scientific, USA).

**Table 2:** All the primers used in this study.

Name of primer	Sequence (5’-3’)	Product size (bp)	Annealing temperature (Jones et al.)	Reference
Nad_F Nad_R	GATGCTTCTTGGGGCTTCTTGTT CTCCAGTCACCAACATTGGCATAA	181	50	(Menzel et al., 2002)
BRNV_1153 BRNV_1154	GCGCACTGAACCCAAGTTTA CAACATCGAATCCCTCAAGC	502	60	(McGavin et al., 2010)
RLMV_CPhF RLMV_CPhR	CGAAACTTYTACGGGGAAC CCTTTGAAYTCTTTAACATCGT	470	60	(Tzanetakis et al., 2007)
RYNV_1752 RYNV_1753	TCCAAAACCTCCCAGACCTAAAAC ATAATCGCAAAAGGCAAGCCAC	350	55	(Jones et al., 2002)
RVCV_3649 RVCV_3648	CCAACAAAGCTGATATWCCAG CCTCATCTAAGTARTCTTCCA	257	55	(Jones et al., 2019)
LCOI490 HCO2198	GGTCAACAAATCATAAAGATATTGG TAAACTTCAGGGTGACCAAAAAATCA	700	47	(Folmer et al., 1994)

For aphid molecular identification, aphid samples amplified with COI primer were sent for Sanger sequencing (Eurofins Genomics, Norway). The purified PCR products were sequenced in both directions. The nucleotide sequences obtained from Sanger sequencing were analyzed using CLC Genomics Workbench 9.5.1 (Qiagen) and identified using the BLAST service against nr/nt database provided by the NCBI.

### 2.4 Phylogenetic tree

A phylogenetic tree was made to study the genetic differences between two aphid species, *A. idaei* and *Am. idaei*. The sequences of these two species, along with closely related species and outgroups, were first aligned using multiple sequence alignment in Geneious Prime software. After that, the tree was generated using the Neighbor-Joining (NJ) method with the Tamura-Nei genetic distance model. To assess the robustness of the tree, bootstrap analysis was conducted with 1000 replicates. The outgroup for the analysis was set to *Empoasca decipiens* (GenBank Accession: OQ381266).

### 2.5 Aphid cultures

Colonies of *A. idaei* and *Am. idaei* were established as individual lines from overwintering eggs collected outdoors in the early spring in 2022 and subsequently tested for viruses. The aphids were kept on virus-free raspberry plants of cv. Glen Ample derived from tissue culture plants. The aphid cultures were maintained in net cages (70 x 50 x 50 cm) in a climate room at 18°C, 75% humidity, and a 16-h light/8-h dark cycle. All experiments were carried out under the same conditions in separate aphid net chambers, i.e., one cage per treatment.

### 2.6 Aphid transmission

Adult wingless aphids from tested BRNV-free colonies of *Am. idaei* and *A. idaei* were employed in the experiment. Starvation time was determined by not feeding the aphids for various periods of time up to two hours and then offering them a raspberry leaf to observe whether feeding commenced immediately or not using a Lecia MZ72 stereomicroscope. Based on this, the starvation time was set to one hour.

**Figure 1:**
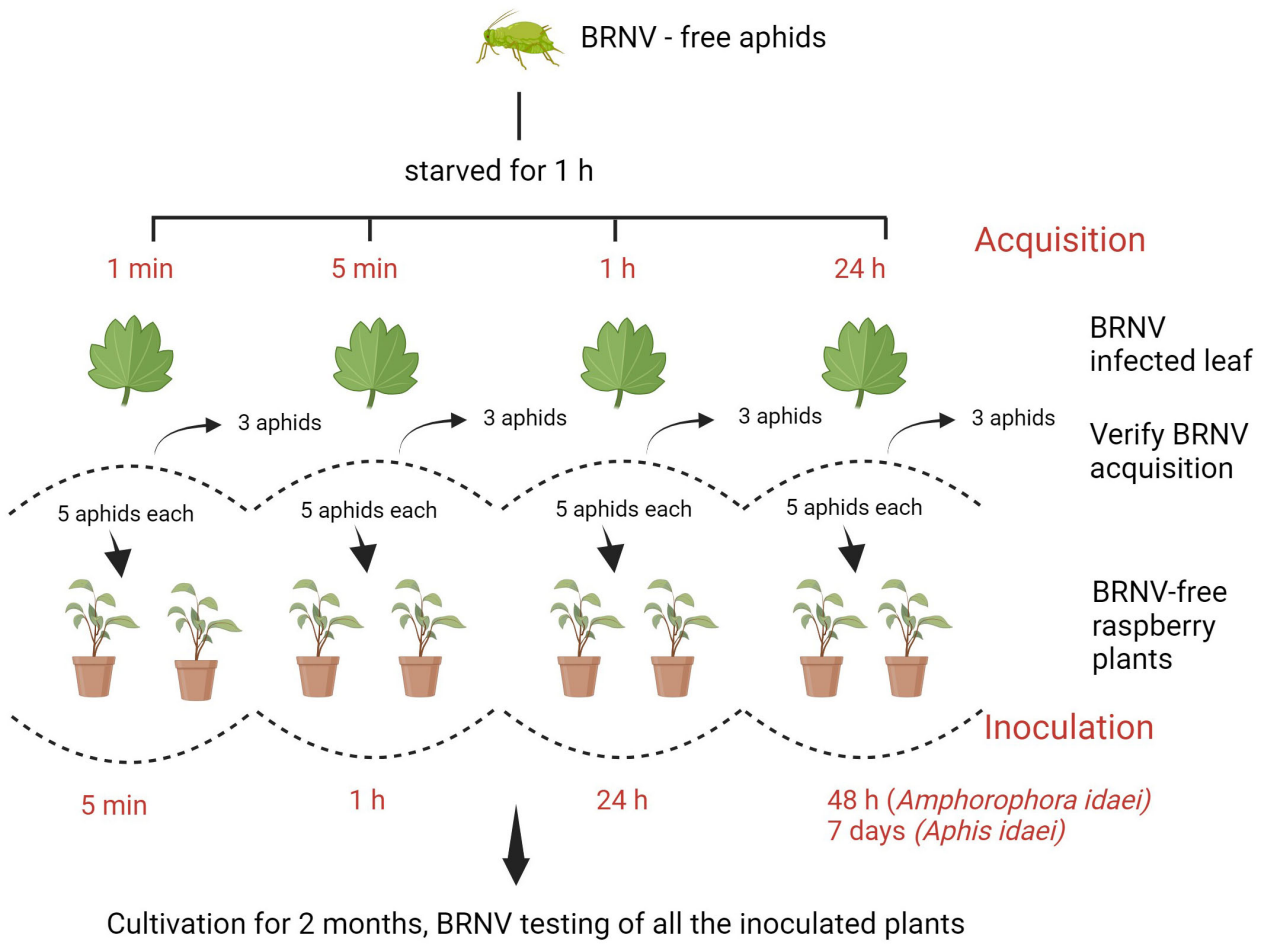
Schematic illustration of the assay for aphid-assisted transmission of black raspberry necrosis virus (BRNV) to raspberry plants. Created with BioRender.com.

The aphid transmission experiment was carried based on methods from Halgren et al. (2007) and Koloniuk et al. (2023), with modifications. For the virus acquisition phase, aphids were allowed to feed on BRNV-infected leaves for different periods of time: 1 minute, 5 minutes, 1 hour, and 24 hours, with each acquisition group consisting of 13 aphids. Following this feeding period, three aphids were immediately tested for virus acquisition and a group of five aphids was carefully placed on the upper surfaces of each of two BRNV-free raspberry plants of raspberry cv. Ninni. The aphids were then placed for specific inoculation periods, including 5 minutes, 1 hour, 24 hours, and 48 hours (for *Am. idaei*) or 7 days (for *A. idaei)* as shown in **Figure 1**. Additionally, the experiment for *A. idaei* was repeated with three individual BRNV-free plants to verify the consistency and reproducibility of the transmission results. In total, five plants were applied for *A. idaei* transmission.

### 2.7 Data analysis

Statistical analyses were performed with R-Studio software, using Microsoft Excel for graphical representation. The maps were created using the QGIS 3.24 software. For this purpose, Norway’s shape file was obtained from Kartverket, the Norwegian mapping authority. The file was accessed via the following link: https://kartkatalog.geonorge.no/metadata/administrative-enheter-fylker/6093c8a8-fa80-11e6-bc64-92361f002671.

## 3 Results

### 3.1 Annual virus occurrence and geographical distribution

**Figure 2:**
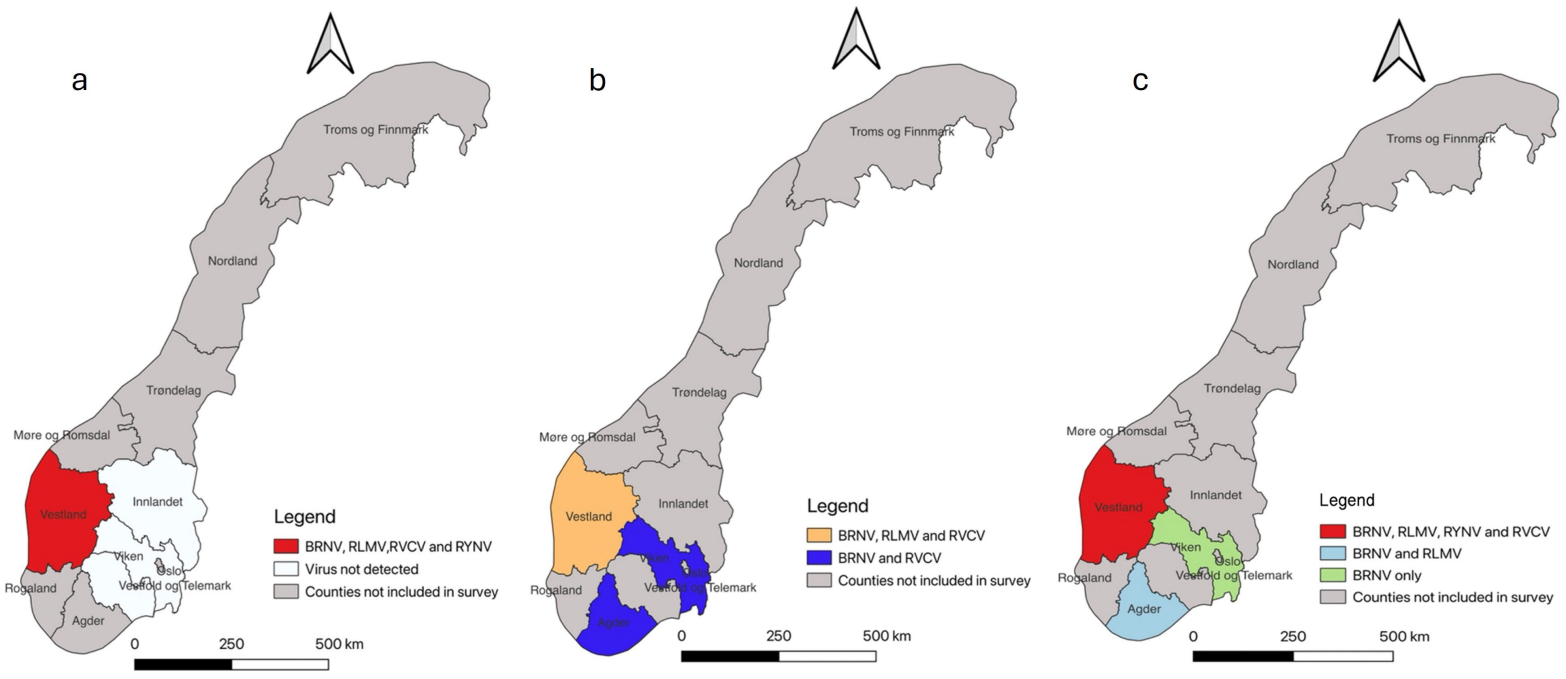
Map of Norway illustrating the surveillance area at county level and virus occurrences detected in each county. The gray color designates the counties not included in our study. **(A)** Survey results from 2019 reveal that all mentioned viruses were exclusively detected in Vestland County. **(B)** Survey results from 2021 indicate the presence of three different viruses in Vestland County (orange): BRNV, RLMV, and RVCV. In two other counties (blue), the detection of two viruses, BRNV and RVCV, is noted. **(C)** Survey results from 2022 indicate that all mentioned viruses were once again found exclusively in Vestland County. In Agder County (light blue), only BRNV and RLMV were detected, while in Viken (light green), only BRNV was present.

In 2019, out of 63 samples tested, 23 were found to be infected with one of the tested viruses. Notably, BRNV dominated with 22 cases, followed by RLMV (4) and RYNV (2). RVCV was detected in only one plant sample. All virus-infected samples were collected in Vestland County, with no occurrences in the other surveyed regions (**Figure 2A**).

In 2021, out of 95 tested leaf samples, 72 exhibited infections with one of the tested viruses. Once again, BRNV was predominant, found in 64 out of 95 samples, followed by RLMV (21) and RVCV (6). Interestingly, no instances of RYNV were detected this year. BRNV, RLMV, and RVCV were identified in samples from Vestland County, while BRNV and RVCV were also found in Viken and Agder Counties (**Figure 2B**).

In 2022, 83 out of 116 tested leaf samples displayed infection with one of the tested viruses. BRNV maintained its dominance with 79 cases, followed by RLMV (17) and RVCV (15). Additionally, RYNV was detected in 10 plant samples this year. Agder County exhibited the presence of only BRNV and RLMV, while Viken County had solely BRNV (**Figure 2C**). All mentioned viruses were identified in Vestland county including RYNV. The overall distribution of mentioned viruses in raspberry cultivars across different Norwegian counties (2019, 2021, and 2022) are tabulated in **Supplementary Table S1**.

### 3.2 Occurrence of single and mixed virus infections

In total, out of 274 collected samples, 178 samples exhibited either sole infection by a single virus or co-infections involving multiple tested viruses (**Table 3**). Upon analyzing the collective data spanning all three years, a clear pattern emerged, highlighting the substantial prevalence of BRNV, with an infection rate of 93.3% (166 out of 178). BRNV single infection accounted for a noteworthy 63.4% of the total infected samples, with 113 instances. Next in prevalence was mixed infections of BRNV and RLMV, constituting 16.2% of the total infected samples, amounting to 29 cases. In contrast, the single infection rate of the other aphid-borne viruses, namely RLMV, RYNV, and RVCV, was notably low (**Table 3**). Examining the co-infection dynamics, it was observed that the co-infection rate of BRNV with RLMV remained consistently high. Moreover, the survey unearthed instances of RMD, arising from concurrent infections of BRNV, RLMV, and RYNV. These instances were relatively scarce, constituting only 2.8% of the total infected samples, with a count of 5 cases (**Table 3**).

**Table 3:** Presence and incidence of detected raspberry viruses in a total of 274 raspberry samples.

Detected virus	No of RT-PCR positive samples	*Percentage (%) of positive samples in total number of infected samples
BRNV alone	113	63.4
RLMV alone	4	2.2
RYNV alone	3	1.6
RVCV alone	5	2.8
**Single virus detected (total)**	125	70.2
BRNV+RLMV	29	16.2
BRNV+RYNV	3	1.6
BRNV+RVCV	12	6.7
**Co-infection with 2 viruses (total)**	44	24.7
BRNV+RLMV+RYNV	5	2.8
BRNV+RLMV+RVCV	3	1.6
**Co-infection with 3 viruses (total)**	8	4.4
BRNV+RLMV+RYNV+RVCV	1	0.5
**Co-infection with 4 viruses (total)**	1	0.5

Total number of samples (n)=274, total infected samples=178.

*Relative occurrence, i.e., percentage derived by dividing the No of RT-PCR positive samples by No of Total infected samples (178).

**Figure 3:**
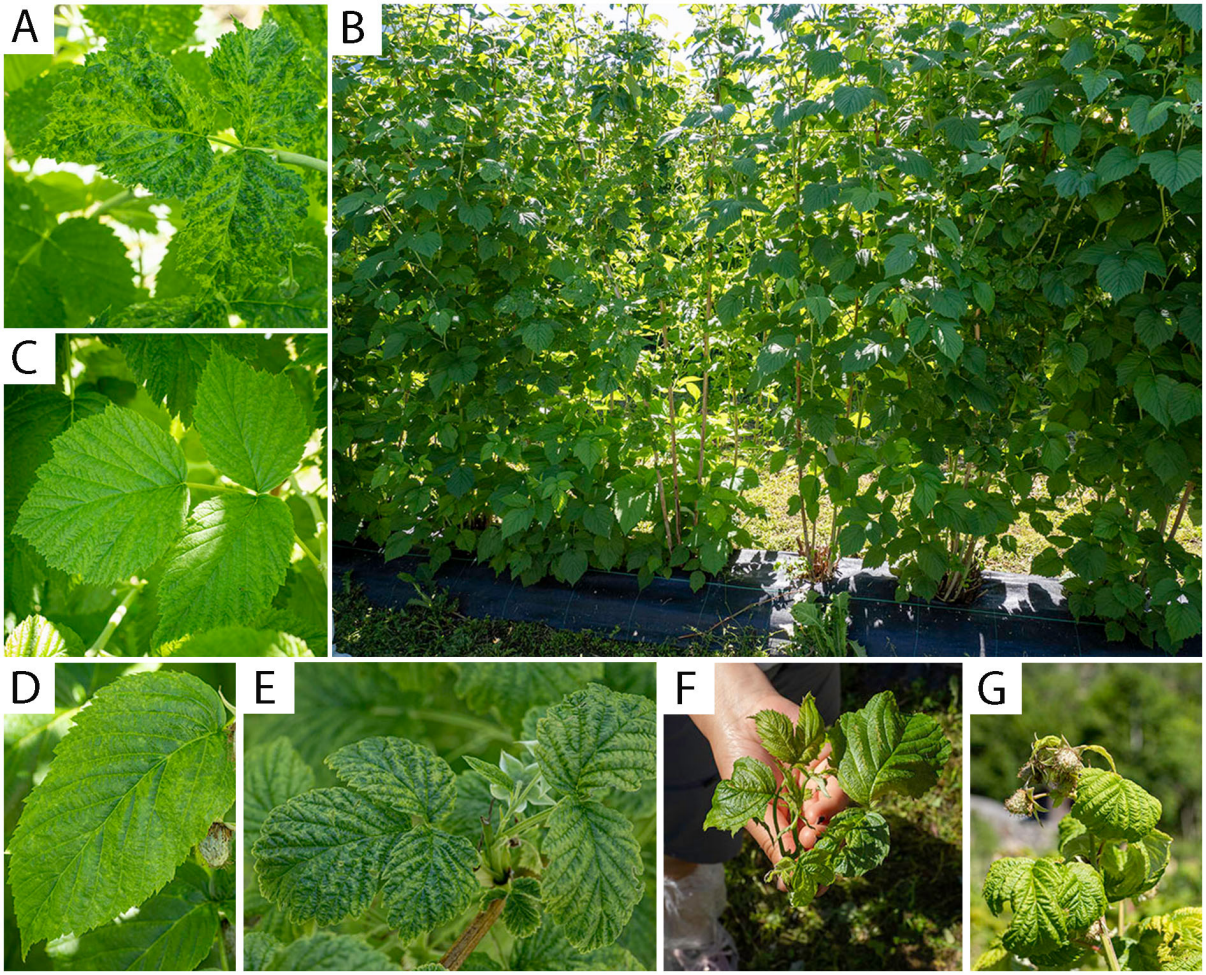
Virus-infected raspberry leaves and plants with diverse symptoms. **(A)** BRNV-infected raspberry cv. Glen Ample leaf with mosaic and vein clearing symptoms; **(B)** BRNV-infected raspberry cv. Glen Ample plants in a row: with heavy mosaic symptoms and reduced plant vigor in the middle of the row and random mosaic symptoms spreading to the neighbor plants in both sides; **(C)** BRNV-infected raspberry cv. Glen Ample leaf with no obvious symptoms; **(D)** BRNV and RLMV-infected raspberry cv. Veten leaf with yellow spot symptoms; **(E)** BRNV and RLMV-infected raspberry “wild” plant leaf with mosaic symptoms; **(F)** Raspberry “wild” plant shoot infected with BRNV, RLMV, RYNV and RVCV with yellowing, leaf curl, dwarf and leaf malformation symptoms; **(G)** Raspberry cv. Veten shoot with co-infection of BRNV, RLMV and RVCV (raspberry mosaic disease, RMD) with leaf curl and yellowing symptoms.

Most of the samples infected with BRNV had mosaic and vein clearing symptoms as shown in **Figure 3A**, while some others had no clear symptoms as in **Figure 3C**. Heavily BRNV-infected raspberry cv. Glen Ample plants showed obvious reduced plant vigor with mosaic symptoms on leaves and symptoms spreading gradually to the neighbor plants in both sides (**Figure 3B**). The mixed infection of BRNV with other virus exhibited more intense symptoms (**Figures 3D–G**) varying from mosaic, yellowing, leaf curl, dwarf, and leaf malformation symptoms.

### 3.3 Virus infection and cultivars

**Figure 4:**
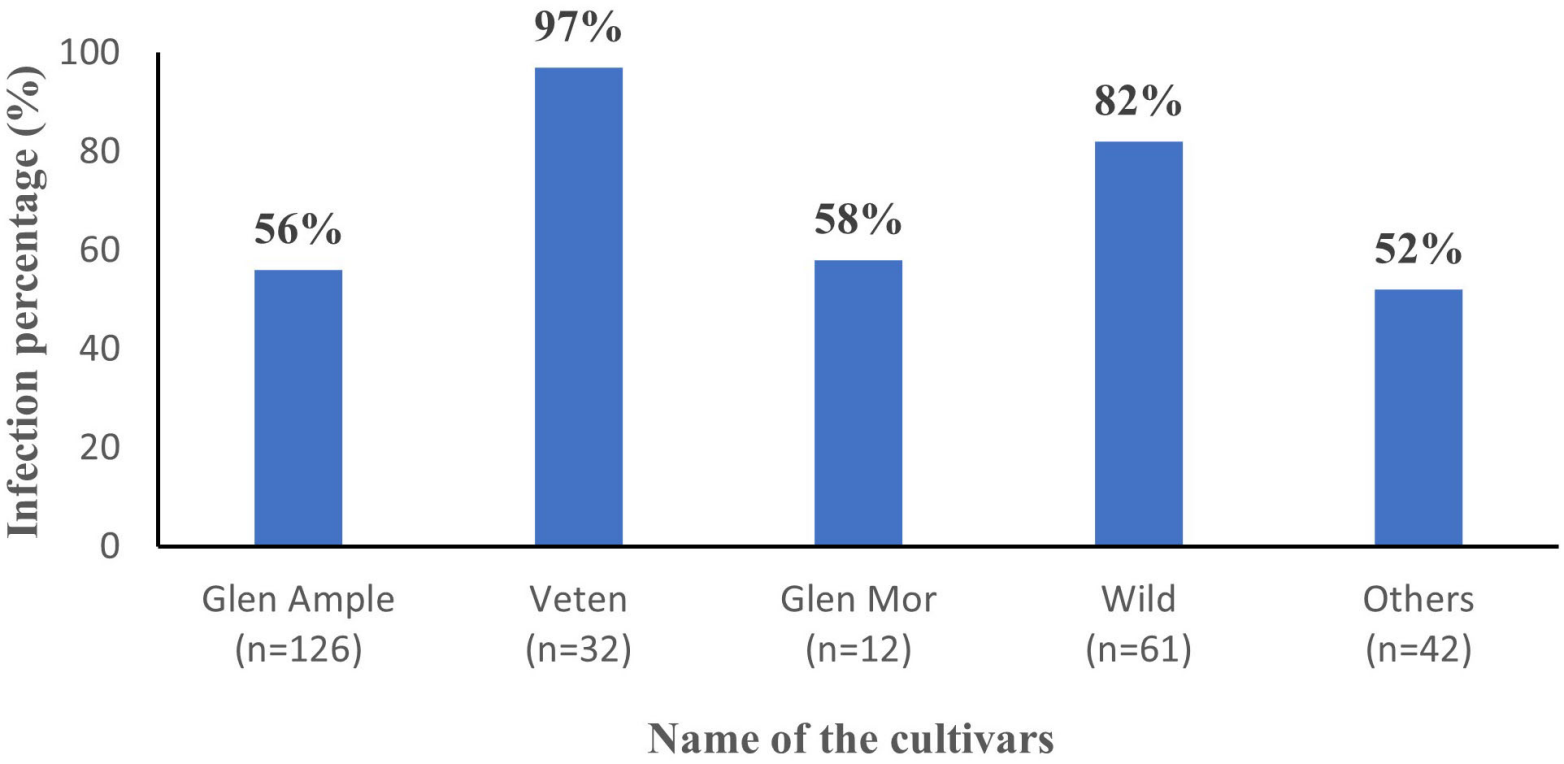
Average virus infection percentage of different cultivars (n= number of samples collected).

**Figure 5:**
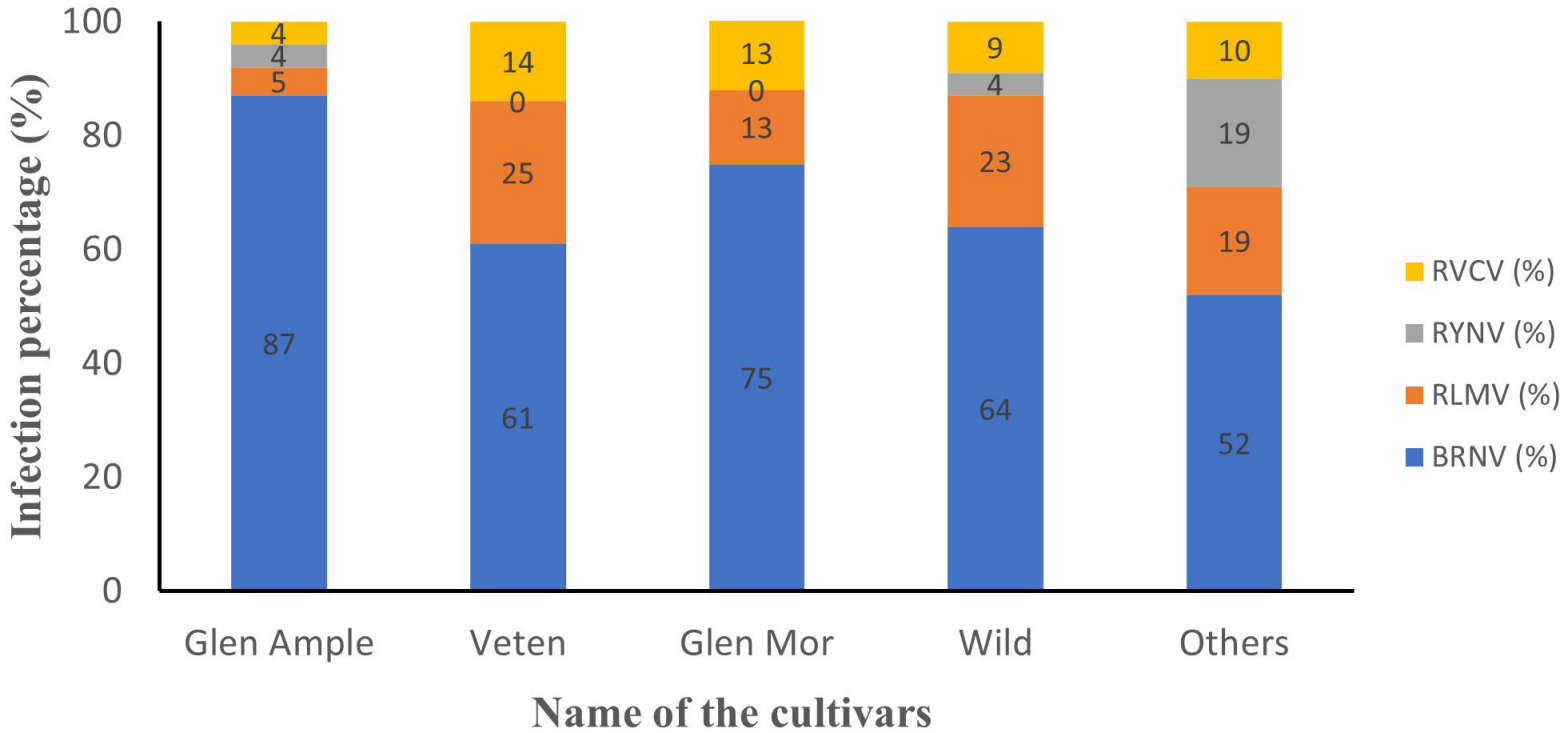
Infection percentage of different viruses within the cultivars. BRNV was dominant in all cultivars. RYNV was not detected in raspberry cv. Veten and cv. Glen Mor.

All three cultivated cultivars (‘Glen Ample’, ‘Glen Mor’, and ‘Veten’), along with the “Wild” and the “Others” category, demonstrated infection by at least one of the tested viruses (**Figure 4**). Notably, cv. Veten samples exhibited the highest infection rate, with 97% (31 out of 32 samples) infected. Following this, uncultivated raspberry samples (“Wild”) showed an infection rate of 82% (50 out of 61 samples), and cv. Glen Mor of 58% (7 out of 12 samples). ‘Glen Ample’ exhibited a virus infection rate of 56% (70 out of 126 samples), and “Others” 52% (22 out of 42 samples). An analysis of virus infestation prevalence within these cultivars revealed that BRNV dominated across all cultivars (**Figure 5**). Notably, RYNV was not detected in the Veten and Glen Mor cultivars.

### 3.4 Aphid species distribution and virus infection

In total, 107 total RNA were extracted from collected aphid samples and checked with RT-PCR using COI primers before conducting virus diagnostics. Molecular identification of aphid species was performed on 13 and 76 aphid samples from 2021 and 2022, respectively. Specifically, only virus-infected aphids were molecularly identified in 2021, whereas all the COI-positive aphids were identified in 2022.

A total of 77 aphids were successfully molecularly identified, with 54 classified as *Am. idaei* and 23 as *A. idaei* (**Supplementary Table S3** and **Table 4**). Amplicons of 575bp with 100% identity and 100% coverage were obtained for *A. idaei* (NCBI accession no. KF638947; 658 bp). Amplicons of 633 bp showed 100% identity and 100% coverage for large blackberry aphid, *Am. rubi* (NCBI accession no. JX507416; 668 bp). Based on the sampling host being raspberry, not blackberry, these amplicons were identified as large raspberry aphid, *Am. idaei*. These two sequences have been submitted to the NCBI GenBank: *Am. idaei* (accession no. PP265263) and *A. idaei* (accession no. PQ384946).

**Table 4:** Virus presence in molecularly identified aphid samples collected from raspberry fields.

Aphid species	Total tested samples	Positive with BRNV only	Positive with RLMV only	Positive with BRNV and RLMV	Total positive sample	Negative from tested viruses
*Amphorophora idaei*	54	16	1	5	22	32
*Aphis idaei*	23	6	0	1	7	16

**Figure 6:**
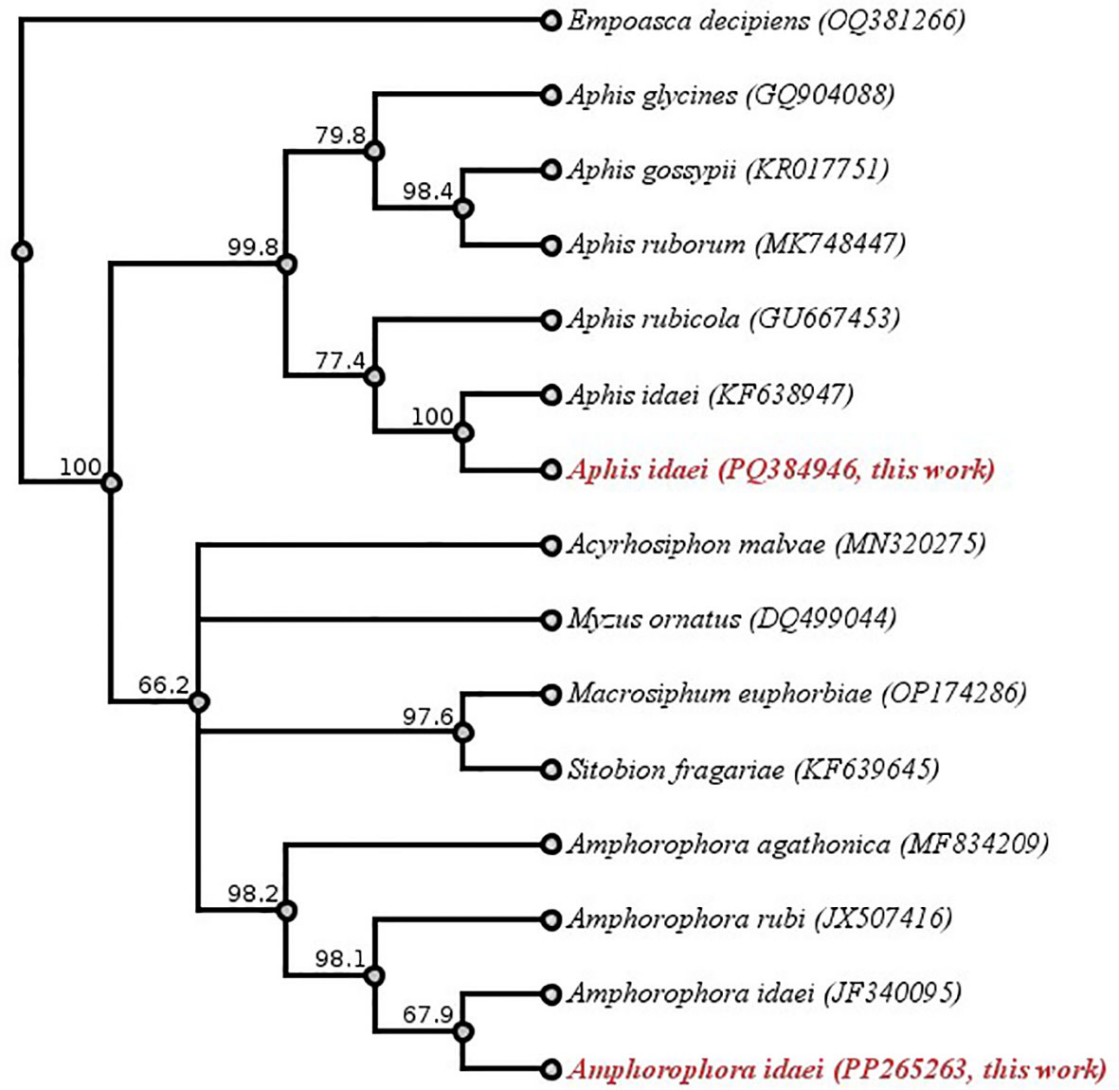
Phylogenetic tree based on COI sequence data for molecular identification of aphids. Species names and GenBank accession numbers are shown at the tips. Sequences highlighted in red, *Aphis idaei* (PQ384946) and *Amphorophora idaei* (PP265263), represent newly obtained sequences from this study. *Empoasca decipiens* (OQ381266) was applied as outgroup. Each branch of the tree contains a bootstrap value. Geneious version 2024.0 created by Biomatters. Available from https://www.geneious.com.

The phylogenetic tree (**Figure 6**) demonstrates a clear genetic distinction between *Am. idaei*, *A. idaei*, and other aphid species, regardless of whether they feed on raspberry plants. This provides molecular evidence for the accurate identification of the collected aphid samples. The phylogenetic relationships shown by the tree are well supported, with most bootstrap values being above 70%, indicating strong confidence in the evolutionary pathway. Here, the tree places *Am. idaei* (this study, GenBank Accession: PP265263) close to the previously available *Am. idaei* sequence (GenBank Accession: JF340095), due to the similarity between the two *Am. idaei* sequences. Both represent the same species, though the shorter sequence (JF340095, 453 bp) may not capture as much genetic variation as the longer sequence obtained in this study (633 bp). In this case, the 633 bp sequence of *Am. idaei* from this study (accession no. PP265263) offers an opportunity to refine these phylogenetic relationships for further study of aphids feeding on raspberry.

In total, one or more viruses were found in 27% of the aphid samples and BRNV was most common in both aphid species (**Supplementary Table S2** and **Table 4**). No aphids were found positive for RVCV or RYNV. In the case of *Am. idaei*, out of the 54 total samples, 16 were found to be positive with BRNV only, 1 for RLMV only, and 5 for both BRNV and RLMV. Notably, 32 samples were free from the tested viruses. For *A. idaei*, comprising 23 samples, 6 were positive for BRNV only, 1 for both BRNV and RLMV, and 16 were free from the tested viruses. The overall distribution of the mentioned viruses in all 107 aphid samples collected in 2021 and 2022 are tabulated in **Supplementary Table S2**.

### 3.5 Transmission assay

**Figure 7:**
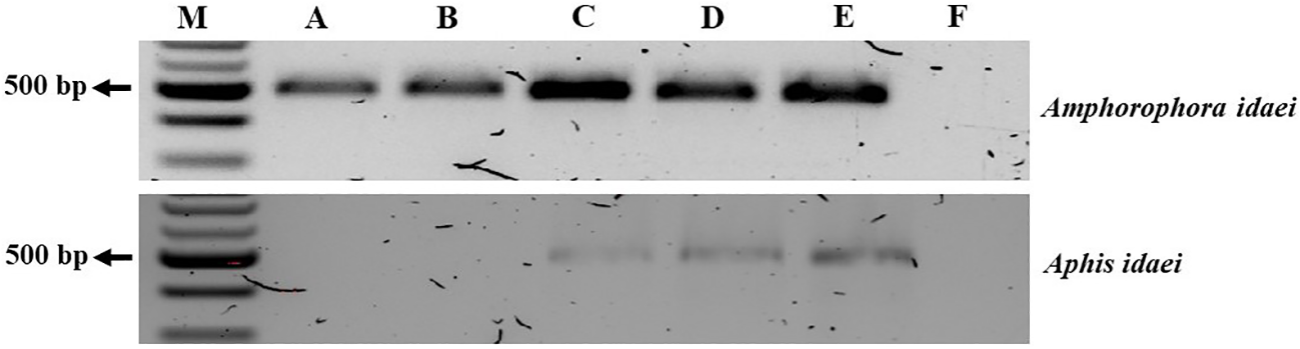
Gel picture of RT-PCR detection results for BRNV in collected aphids after different acquisition periods. From **(A–D)**: aphids collected after 1 min, 5 min, 1 h, and 24 h acquisition times, respectively; **(E)**: BRNV positive control; **(F)**: BRNV negative control; M: 100 bp ladder. The size of the targeted band for BRNV was 502 bp.

**Figure 8:**
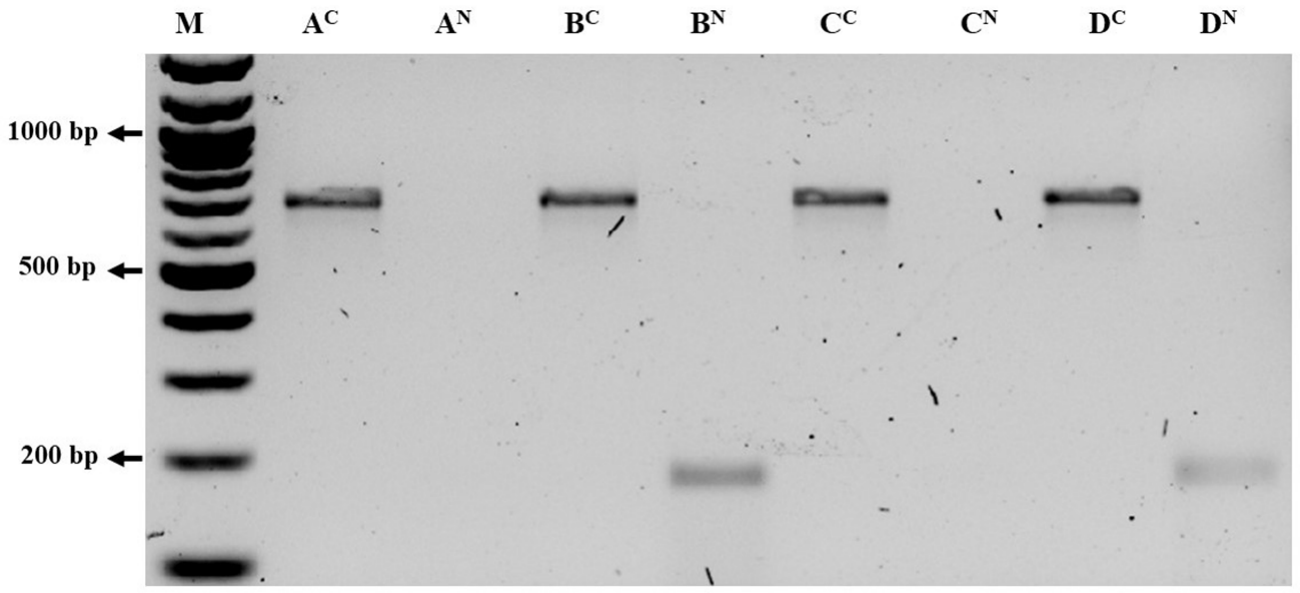
Gel picture of RT-PCR detection results of *Aphis idaei* after different acquisition period using COI and NAD primer. A^c^ and A^N^: COI and NAD primer detection of aphids collected after 1 min respectively; B^c^ and B^N^: COI and NAD primer detection of aphids collected after 5 min respectively_;_ C^c^ and C^N^: COI and NAD primer detection of aphids collected after 1 hours respectively. D^c^ and D^N^: COI and NAD primer detection of aphids collected after 24 hours respectively; M: 100 bp ladder. The size of the targeted band for COI was 700 bp and the size of the targeted band for NAD was 181 bp. Aphids in all acquisition time were positive for COI primer but NAD returned positive for the samples of 5 minute and in 24 hours only.

The aphid cultures underwent initial screening for BRNV using RT-PCR, confirming their BRNV-free status. When feeding aphids with BRNV-infected raspberry leaves for 1 minute, 5 minutes, 1 hour, or 24 hours, *Am. idaei* consistently acquired BRNV and tested positive for BRNV in all the examined acquistion periods (**Figure 7**). Conversely, *A. idaei* aphids did not acquire BRNV after 1 min and 5 min (tested negative for BRNV) but demonstrated BRNV acquistion after 1 hour and 24 hours (tested positive for BRNV) (**Figure 7**). Notably, all BRNV-positive *Am. idaei* aphids tested negative for plant internal *nad5* control, ruling out the presence of plant debris inside the aphids. However, *A. idaei* were positive for plant internal *nad5* control after 24 hours of acquisition but tested negative after 1 hours of acquisition (**Figure 8**).

**Figure 9:**
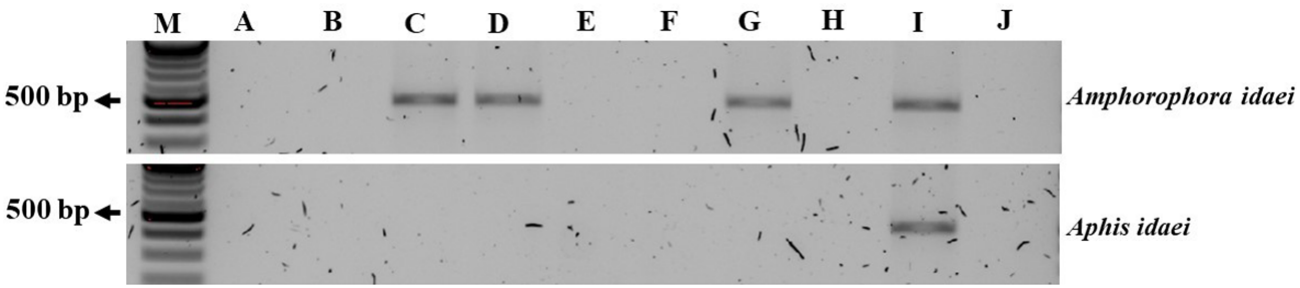
Gel picture of RT-PCR detection results for BRNV in raspberry plants after 2 months of treating BRNV infected aphids in different inoculation periods. From **(A–H)**: raspberry plants tested for BRNV after two month of different inoculation time where, **(A, B)**: plants inoculated for 5 minutes; **(C, D)**: plants inoculated for 1 hours; **(E, F)**: plants inoculated for 24 hours and **(G, H)**: inoculated for 48 hours with *Am. idaei* and 7 days with *A*. *idaei.* Here, **(I)**: BRNV positive control; **(J)**: BRNV negative control; **(M)**: 100 bp ladder. The size of the targeted band for BRNV was 502 bp.

Subsequent testing of all inoculated plants for BRNV using RT-PCR, conducted two months after inoculation, revealed that none of the raspberry plants inoculated by *A. idaei* was positive for BRNV. In contrast, three raspberry plants inoculated by *Am. idaei* were positive for BRNV: two plants from 1- hour inoculation time and one plant from 48 hours (**Figure 9**, **Table 5**).

**Table 5:** Summary of results from BRNV transmission assay with two aphid species.

Aphid species	Acquisition period	RT-PCR test of aphid after acquisition	Inoculation period	RT-PCR test of plants after inoculation	Number of infected plants
*Amphorophora idaei*	1 min.	Pos.	5 min	Neg.	0/2
	5 min	Pos.	1 h	Pos.	2/2
	1 h	Pos.	24 h	Neg.	0/2
	24 hr	Pos.	48 h	Pos.	1/2
*Aphis idaei*	1 min	Neg.	5 min	Neg.	0/5
	5 min	Neg.	1 h	Neg.	0/5
	1 h	Pos.	24 h	Neg.	0/5
	24 h	Pos.	7 days	Neg.	0/5

“Pos.” means RT-PCR positive result and “Neg.” means RT-PCR negative result.

## 4 Discussion

### 4.1 Overview of virus occurrence and distribution in plants and aphids

In this investigation, our focus was on detecting four aphid-transmitted raspberry viruses. BRNV was the most common virus found in plant as well as aphid samples. The predominance of BRNV might be attributed to its efficient transmission by the aphid vector, *Am. idaei*, which acquires and transmits the virus to healthy plants within minutes (Stace-Smith, 1955b). In contrast, RLMV and RYNV exhibit longer acquisition and inoculation times in *Am. idaei*, and the same is the case for RVCV in *A. idaei* (Stace-Smith, 1955a, Stace-Smith, 1961; McMenemy et al., 2009). The high incidence of BRNV in raspberry growing area in Norway is corresponding to the previous survey in Finland (Susi et al., 2018).

Our study revealed a high percentage of co-infection occurrence of BRNV and RLMV both in plants and aphids, a trend aligning with reports of common co-infections in Europe (Converse, 1987). This co-infection is facilitated by the shared aphid vector and potentially also an attraction of the aphid vector to virus-infected plants within a short time window (McMenemy et al., 2012). Moreover, high co-infection rates are to be expected when mainly symptomatic plants are sampled, because BRNV, when found as a single infection, typically does not present symptoms on red raspberry (Stace-Smith, 1955b; Jones and Jennings, 1980). However, the high incidence of BRNV single infections in our study (41% of all our leaf samples) points to a presence of symptoms, and we observed mosaic and vein clearing symptoms on most of the Glen Ample leaf samples with BRNV single infection. None of these samples were found to be infected with either raspberry bushy dwarf virus or the raspberry leaf blotch virus, either. To fulfill Koch’s postulates, transmission of BRNV to virus-free Glen Ample plants to induce similar symptoms should be further pursued. When BRNV was present with other examined viruses, i.e., as co-infections, the resulting symptoms were distinctive (**Figure 3**), as reported in earlier literature (Martin et al., 2013).

### 4.2 Virus infection and cultivars

Among the surveyed raspberry samples, ‘Veten’ exhibited the highest virus infection rate, followed by “wild” raspberries found in the semi-natural vegetation that frequently adjoins Norwegian raspberry crops. ‘Veten’ was prominent in Norway’s raspberry industry for over 30 years, primarily grown for industrial use. However, over the past two decades, it has been replaced by ‘Glen Ample’ (Heiberg et al., 2002), a cultivar with a specific resistance gene, A1, which to a certain extent provides it with a defense against the important virus vector *Am. idaei* (McMenemy et al., 2009). This shift in cultivars must have forced *Am. idaei* populations to prefer the few ‘Veten’ crops left or remain on wild raspberry, resulting in an accumulation of virus infection in these plants. The proliferation of viral infections in “wild” raspberry is a point of concern. These plants may act as viral reservoirs for both pollen-transmitted raspberry viruses like raspberry bushy dwarf virus (not included in this study) and aphid-borne viruses like BRNV (Susi et al., 2018).

The presence of aphid-borne virus in more than half the leaf samples of the two cultivars with some resistance to *Am. idaei*, ‘Glen Ample’ and ‘Glen Mor’, highlights the importance of continued monitoring to reduce the risk of widespread virus transmission even in resistant cultivars and to avoid relying on one method of virus management. *Am. idaei* has been found on ‘Glen Ample’ in Norway since 2015 (Trandem et al., 2015) and was also found in this study; indeed, *Am. idaei* for the transmission experiments was reared on ‘Glen Ample’ without problems. Our study thus aligns with McMenemy et al. (2009), who reported that the *Am. idaei* resistance conferred by the A1 gene had been broadly overcome by other biotypes of the aphid in Scotland, resulting in a notable surge in the occurrence of viruses transmitted by this vector. Two of the viruses known to be transferred by *Am. idaei*, BRNV and RLMV, were also found on newly established ‘Glen Mor’ plants with no obvious virus-like symptoms in our study. However, as the sample size was low and no *Am. idaei* was collected on these plants, we do not know if the viruses came with the planting materials or transmitted by aphids after planting.

### 4.3 Aphid species and BRNV transmission assay

Eight different aphids have been reported sucking on raspberries and transmitting different viruses, i.e., *Am. idaei*, *Am. rubi*, *Am. agathonica*, *A. idaei*, *A. rubicola*, *Macrosiphum euphorbiae, Sitobion fragariae, and Myzus ornatus* (Tan et al., 2022). Two of them, i.e., *Am. idaei* and *A. idaei*, were confirmed by combining morphological and molecular identification in this study. This result showed the dominance of *Am. idaei* and *A. idaei* in Norwegian raspberry; they are by far the two most common aphids in European raspberry (Gordon et al., 1997; McMenemy et al. (2009). The absence of the other six aphids in our samples are explainable: *Am. rubi* is typically associated with blackberry (Alford, 2007), *Am. agathonica* and *A. rubicola* are specific in North America (Blackman and Eastop, 2000; Martin et al., 2013) and the others have alternative hosts and are only occasionally observed on raspberry plants (Gordon et al., 1997; Tan et al., 2022).

It should be mentioned that identifying aphids feeding on raspberry based solely on sequence of COI fragments obtained by PCR is insufficient. Initially, all *Amphorophora* on *Rubus* were grouped as a single species, *Amphorophora rubi* (Kaltenbach). Further studies based on host-plant transfers and morphological studies determined that there were two distinct species: *Amphorophora idaei*, which feeds only on red raspberry, and *Amphorophora rubi*, which feeds only on blackberry (Börner, 1939; Blackman et al., 1977). Therefore, combining host information with morphological and molecular data is crucial for accurate aphid identification. The newly submitted COI sequence of *Am. idaei* from this study (accession no. PP265263) may contribute to more precise molecular identification for future studies on aphids feeding on raspberry.

In the transmission experiments, we found that *Am. idaei* can acquire and transmit BRNV within a remarkably short period, one minute for acquisition and within one hour for inoculation. These results correspond with the earlier research of Stace-Smith (1955b), who observed that this aphid required 15 minutes for virus acquisition and around 2 minutes for transmission. Such behavior was reviewed and classified as a semi-persistent type of virus transmission by McMenemy et al. (2009) and was partially observed in our experiment, although the acquisition time in our study was notably shorter, within 1 minute. This discrepancy in acquisition time could be due to the sensitivity of our RT-PCR methodology, which can detect lower virus concentrations than the traditional indicator-plant based methods used by Stace-Smith (1955b). Furthermore, our experiments revealed that virus transmission was not observed within 5 minutes but only became apparent after 1 hour. It is crucial to recognize that aphid behavior significantly influences transmission rates, and transmission is not always guaranteed, as noted by Stace-Smith (1955b). Additionally, the limited sample size in our study, with only 2 plants per treatment, may have contributed to the absence of observed transmission within the 5-minute inoculation period.

For the transmission experiment with *A. idaei*, the aphids acquired BRNV within 1 hour, but transmission to healthy raspberry plants could not be confirmed. Interestingly, *A. idaei* tested positive for the plant internal *nad*5 control after 24 hours of acquisition but tested negative after just 1 hour of acquisition. This suggests uncertainty in the ability of *A. idaei* to effectively acquire BRNV. The virus uptake by the aphid could be through the gut region or hemocoel, whereas transmission is likely hindered by multiple barriers (Lightle et al., 2012). For persistent or circulative mode of transmission, the virus must be retained in the aphid’s salivary gland and for non-persistent transmission, it would require a certain level of specificity, such as protein interactions, for the virus to bind to the aphid’s stylet (Lightle et al., 2012; Abhinash et al., 2023). In our case, the virus may not be able to reach the salivary glands which should be investigated comprehensively to fully understand the underlying mechanisms of transmission.

## 5 Conclusion

Our study focused on the occurrence and distribution of aphid transmitted viruses on various raspberry cultivars in Norway. The results consistently identified BRNV as the most prevalent virus across all year, constituting 93% of total infected samples. Notably, BRNV was most common as a single infection, followed by mixed infection with RLMV, which is the second most prevalent virus. Other viruses like RVCV and RYNV were considerably less common. Observation revealed distinct mosaic patterns and vein clearing symptoms with BRNV single infection that intensified in co-infections with other viruses. This information could assist farmers in early detection of viral infections.

Virus identification in aphid samples and transmission experiments further elucidated the dynamics of BRNV with aphids. *A. idaei* demonstrated the ability to acquire BRNV within an hour but did not transmit it, in contrast to *Am. idaei*, which rapidly acquired and transmitted the virus within the same timeframe. These findings highlight the intricate nature of aphid-virus interactions and emphasize the urgent need for effective management practices. The observed infection rates across different cultivars underscore the necessity for enhanced surveillance and the adoption of virus-free plant materials as a crucial management strategy. Our study lays a foundation for future research and development of integrated control measures, ensuring the sustainability of raspberry production in the face of evolving viral threats and changing climatic conditions.

## Data Availability

The datasets presented in this study can be found in online repositories. The names of the repository/repositories and accession number(s) can be found in the article/**Supplementary Material**.
