# Supplementary material for: Incidence of aphid-transmitted viruses in raspberry and raspberry aphids in Norway and experiments on aphid transmission of black raspberry necrosis virus
Source: Front Plant Sci. 2024 Nov 1;15:1441145. doi: 10.3389/fpls.2024.1441145 (PMC11563961; doi:10.3389/fpls.2024.1441145)
Supplement: Supplementary file 1 [file DataSheet1.docx]

**Table S1** Annual distribution of aphid-transmitted virus infections in raspberry cultivars

across Norwegian counties (2019, 2021 and 2022).

| **County/Cultivar** | **Sample with detected BRNV** | **Sample with detected RLMV** | **Sample with detected RYNV** | **Sample with detected RVCV** | **Total Positive samples** |
| --- | --- | --- | --- | --- | --- |
| **2019** | **22** | **4** | **2** | **1** | **23** |
| **Innlandet** | **0** | **0** | **0** | **0** | **0** |
| Glen Ample | 0 | 0 | 0 | 0 | 0 |
| **Vestfold og Telemark** | **0** | **0** | **0** | **0** | **0** |
| Glen Ample | 0 | 0 | 0 | 0 | 0 |
| Other | 0 | 0 | 0 | 0 | 0 |
| **Vestland** | **22** | **3** | **2** | **1** | **23** |
| Glen Ample | 18 | 2 | 2 | 1 | 19 |
| Veten | 4 | 1 | 0 | 0 | 4 |
| **Viken** | **0** | **0** | **0** | **0** | **0** |
| Glen Ample | 0 | 0 | 0 | 0 | 0 |
| **2021** | **64** | **21** | **0** | **6** | **72** |
| **Agder** | **6** | **0** | **0** | **1** | **6** |
| Glen Ample | 3 | 0 | 0 | 1 | 3 |
| Other | 0 | 0 | 0 | 0 | 0 |
| Wild | 3 | 0 | 0 | 0 | 3 |
| **Vestland** | **49** | **21** | **0** | **4** | **56** |
| Glen Ample | 9 | 0 | 0 | 0 | 9 |
| Glen Mor | 5 | 1 | 0 | 1 | 6 |
| Other | 5 | 2 | 0 | 1 | 7 |
| Veten | 9 | 5 | 0 | 0 | 10 |
| Wild | 21 | 13 | 0 | 2 | 24 |
| **Viken** | **9** | **0** | **0** | **1** | **10** |
| Glen Ample | 3 | 0 | 0 | 0 | 3 |
| Other | 0 | 0 | 0 | 1 | 1 |
| Wild | 6 | 0 | 0 | 0 | 6 |
| **2022** | **79** | **17** | **10** | **15** | **83** |
| **Agder** | **3** | **1** | **0** | **0** | **4** |
| Glen Ample | 1 | 1 | 0 | 0 | 2 |
| Wild | 2 | 0 | 0 | 0 | 2 |
| **Vestland** | **62** | **16** | **10** | **15** | **65** |
| Glen Ample | 22 | 1 | 1 | 1 | 22 |
| Glen Mor | 1 | 0 | 0 | 0 | 1 |
| Other | 10 | 4 | 6 | 1 | 13 |
| Veten | 17 | 7 | 0 | 8 | 17 |
| Wild | 12 | 4 | 3 | 5 | 12 |
| **Viken** | **14** | **0** | **0** | **0** | **14** |
| Glen Ample | 9 | 0 | 0 | 0 | 9 |
| Other | 1 | 0 | 0 | 0 | 1 |
| Wild | 4 | 0 | 0 | 0 | 4 |
| **Grand Total** | **165** | **41** | **12** | **22** | **177** |

**Table S2** Annual distribution of aphid-transmitted virus infections in aphids across

Norwegian counties (2019 and 2021).

| **County/Aphid species** | **Total collected aphid samples** | **Samples with detected BRNV** | **Samples with detected RLMV** | **Total positive samples** |
| --- | --- | --- | --- | --- |
| **2021** | **31** | **12** | **7** | **13** |
| **Agder** | **9** | **5** | **0** | **5** |
| *A. idaei* | 4 | 4 | 0 | 4 |
| bad sequening | 1 | 1 | 0 | 1 |
| * *A. idaei* | 2 | 0 | 0 | 0 |
| * *Am. idaei* | 2 | 0 | 0 | 0 |
| **Vestland** | **20** | **7** | **7** | **8** |
| *A. idaei* | 1 | 1 | 1 | 1 |
| *Am. idaei* | 6 | 6 | 5 | 6 |
| bad sequening | 1 | 0 | 1 | 1 |
| * *A. idaei* | 9 | 0 | 0 | 0 |
| * *Am. idaei* | 3 | 0 | 0 | 0 |
| **Viken** | **2** | **0** | **0** | **0** |
| * *A. idaei* | 2 | 0 | 0 | 0 |
| **2022** | **76** | **18** | **0** | **18** |
| **Agder** | **5** | **3** | **0** | **3** |
| *A. idaei* | 3 | 2 | 0 | 2 |
| *Am. idaei* | 2 | 1 | 0 | 1 |
| **Vestland** | **57** | **15** | **0** | **15** |
| *A. idaei* | 12 | 0 | 0 | 0 |
| *Am. idaei* | 37 | 15 | 0 | 15 |
| bad sequening | 8 | 0 | 0 | 0 |
| **Viken** | **14** | **0** | **0** | **0** |
| *A. idaei* | 3 | 0 | 0 | 0 |
| *Am. idaei* | 9 | 0 | 0 | 0 |
| bad sequening | 2 | 0 | 0 | 0 |
| **Grand Total** | **107** | **30** | **7** | **31** |

* Aphid samples that were only morphologically identified and not detected with any virus.

**Table S3** Molecular identification of aphids collected in two consecutive years (2021

and 2022) by Sanger sequencing with COI primer.

| **Sample name** | **Year** | **Aphid species** | **Amplicons (bp)** | **Query cover (%)** | **Percentage ident. (%)** | **Accession**  **(GenBank)** |
| --- | --- | --- | --- | --- | --- | --- |
| 132-1 | 2022 | *Am. rubi* | 637 | 100 | 99.53 | JX507416 |
| 133-1 | 2022 | *Am. rubi* | 650 | 100 | 99.69 | JX507416 |
| 133-2 | 2022 | *Am. rubi* | 663 | 98 | 99.69 | JX507416 |
| 134-1 | 2022 | *A. idaei* | 660 | 94 | 98.8 | KF638947 |
| 134-2 | 2022 | *A. idaei* | 660 | 94 | 98.8 | KF638947 |
| 135-1 | 2022 | *Am. rubi* | 637 | 100 | 99.69 | JX507416 |
| 136-1 | 2022 | *A. idaei* | 662 | 97 | 98.76 | KF638947 |
| 137-1 | 2022 | *Am. rubi* | 610 | 100 | 99.51 | JX507416 |
| 138-1 | 2022 | *Am. rubi* | 626 | 100 | 99.68 | JX507416 |
| 138-2 | 2022 | *Am. rubi* | 642 | 100 | 99.38 | JX507416 |
| 139-1 | 2022 | *A. idaei* | 659 | 98 | 98.61 | KF638947 |
| 139-2 | 2022 | *A. idaei* | 661 | 98 | 98.92 | KF638947 |
| 140-1 | 2022 | *Am. rubi* | 620 | 100 | 99.52 | JX507416 |
| 140-2 | 2022 | *Am. rubi* | 692 | 96 | 99.55 | JX507416 |
| 142-1 | 2022 | *A. idaei* | 660 | 100 | 98.64 | KF638947 |
| 142-2 | 2022 | *A. idaei* | 661 | 97 | 98.91 | KF638947 |
| 145-1 | 2022 | *Am. rubi* | 639 | 100 | 99.69 | JX507416 |
| 145-2 | 2022 | *Am. rubi* | 650 | 97 | 99.21 | JX507416 |
| 156-1 | 2022 | *A. idaei* | 662 | 100 | 98.64 | KF638947 |
| 160-1 | 2022 | *Am. rubi* | 640 | 100 | 99.69 | JX507416 |
| 160-2 | 2022 | *Am. rubi* | 642 | 100 | 99.69 | JX507416 |
| 162-1 | 2022 | *A. idaei* | 659 | 98 | 98.61 | KF638947 |
| 163-1 | 2022 | *Am. rubi* | 649 | 100 | 99.69 | JX507416 |
| 164-1 | 2022 | *A. idaei* | 664 | 97 | 98.14 | KF638947 |
| 165-1 | 2022 | *Am. rubi* | 633 | 100 | 98.84 | JX507416 |
| 165-2 | 2022 | *Am. rubi* | 641 | 100 | 99.53 | JX507416 |
| 167-1 | 2022 | *Am. rubi* | 674 | 96 | 99.23 | JX507416 |
| 167-2 | 2022 | *Am. rubi* | 632 | 100 | 99.68 | JX507416 |
| 170-2 | 2022 | *Am. rubi* | 638 | 100 | 99.69 | JX507416 |
| 171-1 | 2022 | *Am. rubi* | 632 | 100 | 99.84 | JX507416 |
| 171-2 | 2022 | *Am. rubi* | 654 | 100 | 99.85 | JX507416 |
| 172-1 | 2022 | *Am. rubi* | 643 | 100 | 99.53 | JX507416 |
| 172-2 | 2022 | *Am. rubi* | 653 | 98 | 99.53 | JX507416 |
| 173-1 | 2022 | *Am. rubi* | 663 | 99 | 99.85 | JX507416 |
| 173-2 | 2022 | *Am. rubi* | 671 | 98 | 99.25 | JX507416 |
| 176-2 | 2022 | *Am. rubi* | 657 | 99 | 99.69 | JX507416 |
| 179-1 | 2022 | *A. idaei* | 660 | 98 | 99.23 | KF638947 |
| 183-1 | 2022 | *Am. rubi* | 641 | 100 | 99.69 | JX507416 |
| 186-1 | 2022 | *A. idaei* | 661 | 97 | 98.45 | KF638947 |
| 186-2 | 2022 | *Am. rubi* | 657 | 99 | 99.39 | JX507416 |
| 194-1 | 2022 | *Am. rubi* | 650 | 99.69 | 100.0 | JX507416 |
| 195-1 | 2022 | *Am. rubi* | 640 | 99 | 99.69 | JX507416 |
| 195-2 | 2022 | *Am. rubi* | 641 | 100 | 99.69 | JX507416 |
| 202-1 | 2022 | *Am. rubi* | 640 | 100 | 99.69 | JX507416 |
| 202-2 | 2022 | *Am. rubi* | 646 | 97 | 99.68 | JX507416 |
| 203-1 | 2022 | *Am. rubi* | 641 | 100 | 99.69 | JX507416 |
| 203-2 | 2022 | *Am. rubi* | 625 | 100 | 99.84 | JX507416 |
| 208-1 | 2022 | *Am. rubi* | 631 | 100 | 99.68 | JX507416 |
| 214-2 | 2022 | *Am. rubi* | 643 | 99 | 99.69 | JX507416 |
| 219-1 | 2022 | *Am. rubi* | 633 | 100 | 99.68 | JX507416 |
| 219-2 | 2022 | *Am. rubi* | 632 | 71.7 | 100.0 | JX507416 |
| 220-1 | 2022 | *A. idaei* | 660 | 95 | 98.7 | KF638947 |
| 220-2 | 2022 | *A. idaei* | 620 | 96 | 99.0 | KF638947 |
| 220-3 | 2022 | *A. idaei* | 664 | 99 | 98.79 | KF638947 |
| 222-1 | 2022 | *A. idaei* | 661 | 98 | 98.47 | KF638947 |
| 222-2 | 2022 | *A. idaei* | 661 | 99 | 98.79 | KF638947 |
| 223-1 | 2022 | *Am. rubi* | 643 | 100 | 99.53 | JX507416 |
| 223-2 | 2022 | *A. idaei* | 659 | 97 | 98.44 | KF638947 |
| 240-1 | 2022 | *Am. rubi* | 645 | 100 | 99.53 | JX507416 |
| 240-2 | 2022 | *Am. rubi* | 625 | 99 | 99.68 | JX507416 |
| 241-1 | 2022 | *Am. rubi* | 624 | 100 | 99.68 | JX507416 |
| 241-2 | 2022 | *Am. rubi* | 632 | 100 | 99.68 | JX507416 |
| 245-1 | 2022 | *Am. rubi* | 651 | 100 | 99.54 | JX507416 |
| 245-2 | 2022 | *Am. rubi* | 647 | 100 | 99.69 | JX507416 |
| 246-1 | 2022 | *Am. rubi* | 632 | 100 | 99.53 | JX507416 |
| 246-2 | 2022 | *Am. rubi* | 648 | 100 | 98.77 | JX507416 |
| 14 | 2021 | *A. idaei* | 580 | 100.0 | 99.6 | KF638947 |
| 22 | 2021 | *A. idaei* | 613 | 98 | 98.37 | KF638947 |
| 23 | 2021 | *A. idaei* | 607 | 100.0 | 99.34 | KF638947 |
| 67 | 2021 | *Am. rubi* | 617 | 99 | 98.69 | JX507416 |
| 76 | 2021 | *Am. rubi* | 629 | 100 | 99.52 | JX507416 |
| 77 | 2021 | *Am. rubi* | 630 | 100 | 99.21 | JX507416 |
| 78 | 2021 | *Am. rubi* | 637 | 100 | 99.37 | JX507416 |
| 82 | 2021 | *Am. rubi* | 634 | 100 | 99.05 | JX507416 |
| 96 | 2021 | *A. idaei* | 572 | 99 | 99.83 | KF638947 |
| 60 | 2021 | *Am. rubi* | 612 | 100 | 99.67 | JX507416 |
| 89 | 2021 | *A. idaei* | 580 | 100 | 99.66 | KF638947 |
